# Supplementary figures and images for: Attitudes of the Public to Receiving Medical Care during Emergencies through Remote Physician–Patient Communications
Source: Int J Environ Res Public Health. 2020 Jul 20;17(14):5236. doi: 10.3390/ijerph17145236 (PMC7400122; doi:10.3390/ijerph17145236)

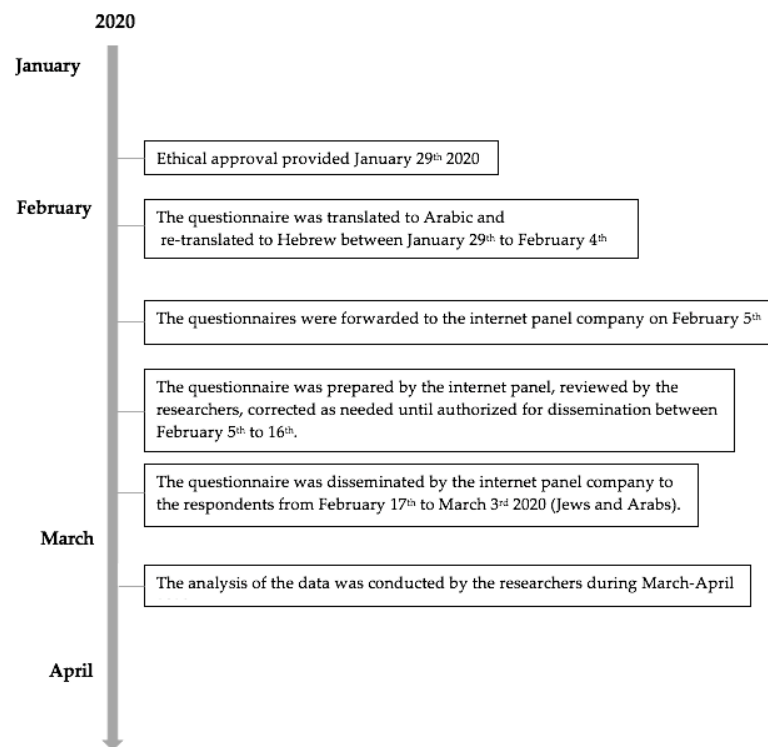

**Figure S1.** Timeline of study procedures.

Supplement: Supplementary file 1 [file ijerph-17-05236-s001.pdf]
